# Metastasis of endometrial adenocarcinoma masquerading as a primary rectal cancer: A rare case report with literature review

**DOI:** 10.1097/MD.0000000000036170

**Published:** 2023-11-17

**Authors:** Minhua Li, Weiping Zheng

**Affiliations:** a Department of Pathology, Shaoxing, Zhejiang, China; b Department of Gynecology, Shaoxing People’s Hospital, Shaoxing, Zhejiang, China.

**Keywords:** endometriosis, metastatic endometrial adenocarcinoma, primary rectal cancer

## Abstract

**Rationale:**

The majority of rectal malignancies are primary tumors, secondary tumors are unusual. The rectal metastasis of endometrial carcinoma is reported to be extremely rare, especially in the absence of endometriosis.

**Patient concerns:**

Herein we present a rare case of a 68-year-old postmenopausal woman with a history of endometrial adenocarcinoma, metastasizing to the rectum 5 years after a hysterectomy and bilateral salpingo-oophorectomy treatments with pelvic lymphadenectomy were performed.

**Diagnoses:**

Histological examination of the rectal neoplasm revealed an invasive lesion in submucosal and muscular layers without definitely invaded evidence in the serous membrane and there was also no obvious endometriosis. The results of immunohistochemistry showed the cancer cells were positive for CK7, estrogen receptor, progesterone receptor, and negative for CK20, villin, confirming the diagnosis of metastatic rectal adenocarcinoma originating from uterine endometrial adenocarcinoma. Meanwhile, the results of immunohistochemical staining showed positive expression of MSH2, MSH6, and negative expression of MLH1 and PMS2, hinting at microsatellite instability which may be related to Lynch syndrome.

**Interventions:**

The Dixon operation with lymph node dissection was performed. Chemotherapy was also performed on this patient for the next 6 months.

**Outcomes:**

The patient was followed up for the next 6 months after surgery and no recurrence was documented until now.

**Lessons subsections:**

Though rectal and endometrial adenocarcinoma could share some similar morphologic features, different immunohistochemical profiles could be revealed between them. Most endometrial carcinoma in the colon or rectum develop from endometriosis. Secondary rectal cancer with endometrial origination in the absence of endometriosis and serosal implants was extremely rare. Therefore, we should pay more attention to this rare but possible presentation for appropriate diagnosis and treatment of these patients.

## 1. Introduction

Endometrial carcinoma is one of the most common gynecologic malignancy. Currently, molecular characterization play a more and more important role in the application of systemic targeted therapy when treating the advanced disease, further increasing overall survival of the patient with endometrial carcinoma.^[1,2]^ After the first treatment of endometrial carcinoma, about 64% of recurrences will present in the next 2 years.^[3]^ Majority of recurrence or metastatic endometrial carcinoma occurs through local extension. The most commonly involved sites were liver, lungs, bones, brain, and peritoneal cavity. The rectum remains an extremely rare metastization site of endometrial carcinoma, which usually associated with endometriosis rather than hematogenous or lymphatic metastasis. This case illustrated the scarcity and peculiarity of metastatic rectal carcinoma originating from uterine endometrial adenocarcinoma without the endometriosis. Moreover, microsatellite instability (MSI) was confirmed in this case, which providing the possibility of targeted therapy. To our knowledge, only 5 similar cases have been published in the literature.^[4–8]^ This is the first case report of endometrial adenocarcinoma with MSI, which metastasis to the rectum in the absence of endometriosis.

## 2. Case report

A 68-year-old Chinese postmenopausal female was admitted to our hospital with a chief complaint of persistent diarrhea and hematochezia for more than half a month. At physical examination, abdominal tenderness was unremarkable. Digital rectal examination did not revealed any mass at the lower part of the rectum. Blood work revealed the level of white blood cells and hemoglobin was similar to the normal baseline. Laboratory tests showed carbohydrate antigen 199, carcinoembryonic antigen level, and other tumor markers were normal as well.

Her past medical history was significant for hypertension for nearly 20 years. More importantly, 5 years prior to the current presentation, she experienced irregular vaginal bleeding for 2 months. Then, she was admitted to outside hospital because uterine cavity occupation was revealed by ultrasonography examination. Finally, total laparoscopic hysterectomy with bilateral salpingo-oopherectomy for endometrial adenocarcinoma was performed in outside hospital. Histological examination of the uterus revealed moderately endometrial adenocarcinoma, endometrioid type, Federation International of Gynecology and Obstetrics grade I–II. She was determined to have stage IA disease (T1b N0 M0) that invaded less than half of the myometrial thickness. Radiotherapy were also performed in this patient during the next 5 years. However, pan-computed tomography was negative for any obvious recurrence and distant metastasis of endometrial carcinoma in our hospital.

**Figure 1.**
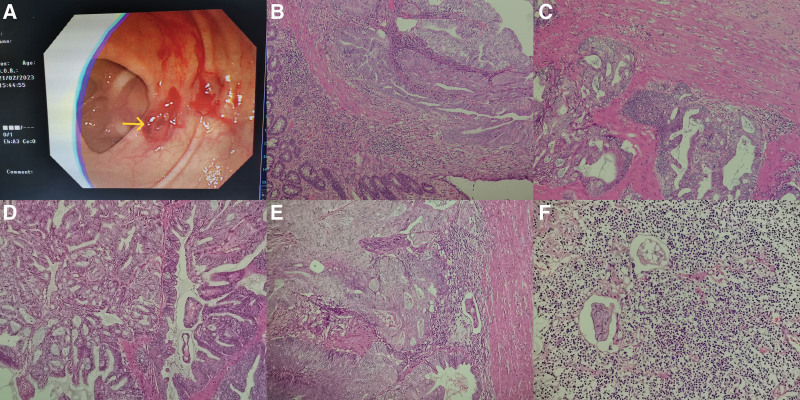
(A) Colonoscopy revealed a ulcerated mass with irregular elevations at the margins in the rectum. (B) Atypical neoplastic cells were showed in submucosal of rectal (×200). (C) The muscular layer of rectum was invaded and destroyed by the carcinoma cells (×200). (D) The tumor was composed of large numbers of glandular epithelium with cribriform or papillary glandular architecture (×200). (E) The vessel invasion was showed in part of the lesion (×200). (F) Focally, lymph node metastasis was revealed (×200).

Then, she underwent a colonoscopy for further exploring the cause of persistent diarrhea and hematochezia. The result revealed a 2 cm ulcerated mass with irregular elevations at the margins and accounted for one-third of the circumference of the lumen in the rectum accompanied with multiple polyps in transverse colon (Fig. 1A). Histological examination of biopsies was performed on the ulcerated mass in the rectum. The results showed unequivocal features of adenocarcinoma without visible goblet cells in the rectum. Therefore, the Dixon’ operation with lymph node dissection was performed. The mass was presented in the upper part of rectum, measuring 18 * 15 mm.

**Figure 2.**
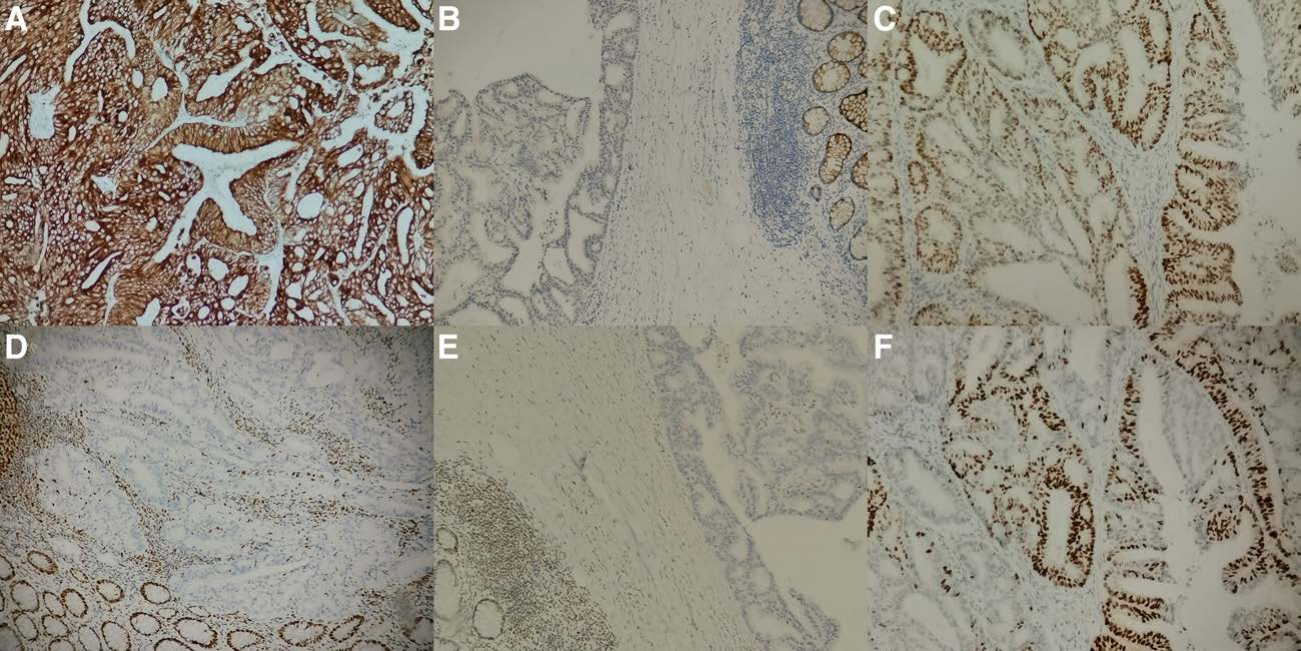
(A) Definitely positive staining of ER was present in tumor cells (×200). (B) Positive expression of PR was evident (×200). (C) Strong staining of CK7 was displayed (×200).(D) Negative staining of CK20 was showed (×200). (E) Completely negative expression of MLH1 were revealed (×200). (F) Negative staining of PMS2 was found in the tumor cells (×200). ER = estrogen receptor, PR = progesterone receptor.

Histological examination of the neoplasm revealed an invasive lesion in submucosal and muscular layer without definitely invaded evidence in the serous membrane (Fig. 1B and C). Moreover, there were not any obvious endometriosis in the rectum as well. The tumor was composed of large numbers of glandular epithelium with cribriform or papillary glandular architecture, mimicking a primary rectal adenocarcinoma (Fig. 1D). Obvious moderate nuclear atypia and abnormal mitoses is frequently seen. However, there were lack of definite goblet cells and there was not obvious squamous differentiation features. The vessel invasion was showed in part of the lesion and lymph node metastasis was revealed as well (Fig. 1E and F). The results of immunohistochemistry showed the tumor cells were positive for CK7, estrogen receptor, progesterone receptor, and negative for CK20, Villin (Fig. 2A–D). Simultaneously, MLH1, PMS2 were negative and MSH2, MSH6 were positive (Fig. 2E and F), hinting MSI which may be related to Lynch Syndrome (LS). Therefore, the we made a diagnosis of metastatic moderately endometrial adenocarcinoma in rectum.

Chemotherapy were also performed in this patient for next 6 months and no recurrence was documented until now.

## 3. Discussion

The incidence of endometrial cancer has been rapidly increasing along with the higher rates of obesity, which is the most important risk factor for this disease. Though endometrial cancer could give rise to hematogenous or lymphatic metastasis, most of them usually presented in liver, lungs, bones, brain, and peritoneal cavity. Rectum was the unusual metastasis site for endometrial cancer. Morphologically, the rectal adenocarcinoma in our case more closely resembled a primary rectal cancer compared with metastasized cancer. Thus far, only 5 similar cases have been published in the literature. Recently, 2 rare similar cases of sigmoid colonic metastasis of endometrial adenocarcinoma in the absence of endometriosis was illustrated.^[4,8]^ To date, no case was reported in rectum, especially in the patient with MSI. Therefore, this is the first case report of metastasized endometrial adenocarcinoma occurring in rectum without endometriosis.

Endometrioid adenocarcinoma was the most common histologic type of endometrial cancer, which accounting for 80% of all endometrial carcinomas.^[3]^ Recurrence or metastasis of endometrial cancer occurring in intra-abdominal organs usually present in aggressive histopathologic types such as the serous and clear cell type. Endometrial cancer with higher stages have more propensity for direct invasion of surrounding organs and lymphatic or hematogenous metastasis. Age > 60 years, histologic grade 3, myometrial invasion > 50%, lower uterine involvement and lymphovascular invasion were the risk factors for colonic metastasis from endometrial adenocarcinoma.^[5]^ In our case, typical endometrioid adenocarcinoma with grade 1–2, myometrial invasion < 50%, lack of lymphovascular invasion was revealed. Therefore, we should pay more attention on the endometrial cancer with less aggressive histopathologic or lower stages as well.

Secondary cancers of the rectum frequently result from peritoneal seeding or direct spread. So serosal implants often present in the rectum.^[6]^ In our case, intraluminal mass was seen without invaded evidence in the serous membrane, resembling a primary rectal cancer. This is a an extremely rare phenomenon in the secondary cancers of the rectum. Meanwhile, most documented cases of endometrial carcinoma in colon or rectum developed from endometriosis. In our case, owing to the lack of endometriosis and serosal implants, it has been proposed that hematogenous or lymphatic dissemination may play an important role. Moreover, we found the existence of vessel invasion, further confirming the possibility of hematogenous dissemination.

Morphologically, rectal and endometrial adenocarcinoma could share some similar morphologic features. However, goblet cells were more frequently seen in the cancer with gastrointestinal origination. Our case showed the lack of definite goblet cells, hinting the impossibility of gastrointestinal origination. One the other hand, squamous differentiation features often present in endometrial adenocarcinoma. No obvious squamous differentiation features was revealed in this case, further confirming the possibility of endometrial adenocarcinoma. Immunohistochemistry staining played an important role in distinguishing the endometrial adenocarcinoma from primary rectal adenocarcinoma. Tumor cells in our case were positive for CK7, estrogen receptor, and progesterone receptor, which frequently present in female reproductive tract or breast tumor.CK20 and Villin were the important marker of intestinal tract tumor. Negative expression of CK20 and Villin were also showed in this case. All of them fully confirmed the diagnosis of metastatic rectal adenocarcinoma originating from uterine endometrial adenocarcinoma.

In terms of pathogenesis of endometrial cancer, part of them has confirmed been linked to MSI, which may be associated with LS. As a hereditary nonpolyposis colorectal cancer syndrome, LS is an autosomal dominant disease.^[9]^ The pathogenesis of LS is the mutation of mismatch repair gene, including MLH1, MSH2, MSH6, and PMS2. Duplicated coding gene fragment or noncoding gene fragment may mutate when the function of mismatch repair genes are lost, resulting in MSI.^[9]^In consideration of the negative expression of MLH1,PMS2, LS was not excluded in our case. Unfortunately, the gene testing was not carried out due to the financially problem. However, given the lack of familiar history and age of this patient, this case was prone to a sporadic adenocarcinoma.^[1,10]^

## 4. Conclusions

In a conclusion, we present a rare case report of secondary rectal cancer with endometrial origination in the absence of endometriosis, masquerading as a primary rectal cancer in histological morphology. However, different immunohistochemical profiles could be revealed between endometrial adenocarcinomas and primary rectal carcinomas. Therefore, we should not ignore the rare possibility of metastatic rectal adenocarcinoma originating from uterine endometrial adenocarcinoma, even if there is no endometriosis.

## Author contributions

**Investigation:** Minhua Li.

**Project administration:** Weiping Zheng.

**Writing – original draft:** Minhua Li.

**Writing – review & editing:** Weiping Zheng.
